# Cardiotoxicity occurred in aged C57BL/6nia mice given a formulation containing poloxamer 407 hydrogel

**DOI:** 10.1080/20010001.2017.1304004

**Published:** 2017-03-30

**Authors:** Terri Iwata, John Morton, Jessica Snyder, Xuan Ge

**Affiliations:** ^a^ Department of Comparative Medicine, University of Washington, Seattle, WA98195, USA

**Figure 1.**
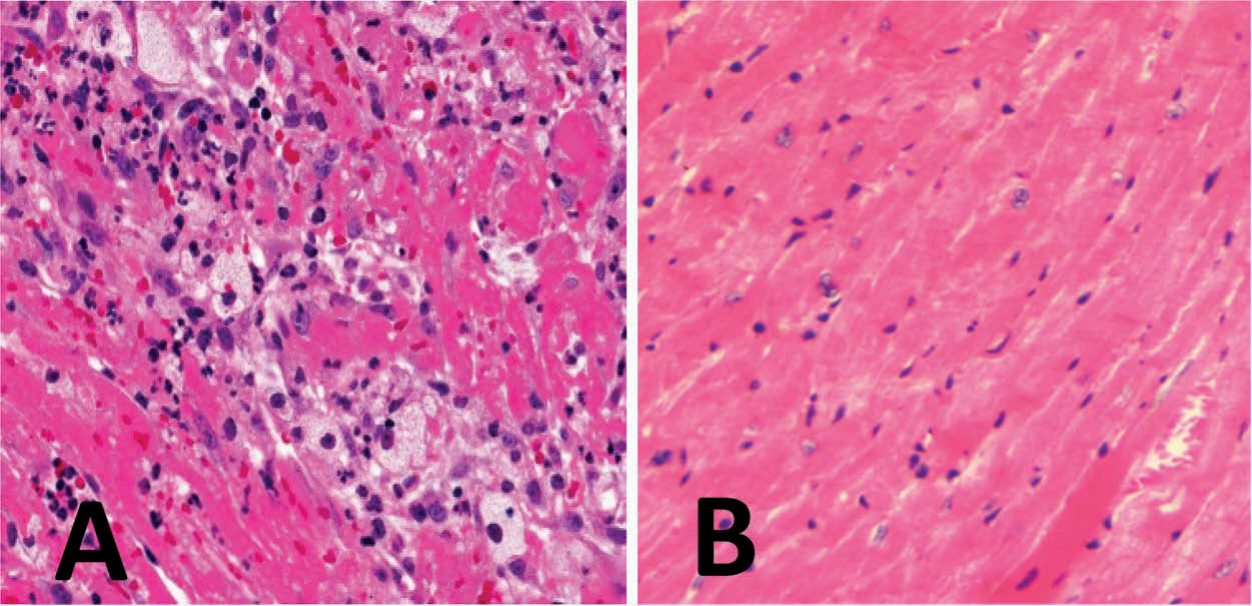
(A) Cardiac lesions in a 24-month-old C57BL/6nia male mouse treated with a poloxamer 407 hydrogel formulation were characterized by moderate to severe necrotizing myocarditis, histiocytes with abundant vacuolated cytoplasm, and hemorrhage. (B) For comparison, an essentially normal heart from an age- and gender-matched mouse not exposed to the poloxamer 407 formulation is shown. Myocardial necrosis and inflammation are not considered age-related lesions.

Poloxamer 407 is a thermoreversible hydrogel frequently used as a carrier for polymer formulations in delayed-absorption drug studies, with limited toxicity in young mice. We gave six 24-month-old C57BL/6nia male mice each a subcutaneous injection of polylactic acid (PLA)/polyglycolic acid (PGA) copolymer biodegradable microparticles mixed with poloxamer 407 at a dose of 1.1 g/kg and 6 g/kg, respectively. Six days following injection, the animals presented as mild to moderately lethargic and dehydrated, and were subsequently killed. Histological evaluation revealed moderate to marked necrotizing myocarditis and myocardial hemorrhage (Figure 1). Two additional cohorts of similarly aged C57BL/6nia mice received the same PLA/PGA microparticle formulation but without poloxamer 407, and showed no adverse clinical signs or lesions of necrotizing myocarditis. These observations suggest that aged C57BL/6nia mice can be sensitive to the cardiotoxic effects of poloxamer 407 under certain dose conditions.

